# Safety of Newer Disease Modifying Therapies in Multiple Sclerosis

**DOI:** 10.3390/vaccines9010012

**Published:** 2020-12-26

**Authors:** Georges Jalkh, Rachelle Abi Nahed, Gabrielle Macaron, Mary Rensel

**Affiliations:** 1 Department of Neurology, Faculty of Medicine, Université Saint Joseph, Beirut B.P. 11-5076, Lebanon; georges.jalkh@net.usj.edu.lb (G.J.); rachelle.abinahed@net.usj.edu.lb (R.A.N.); gabrielle.macaron@hdf.usj.edu.lb (G.M.); 2 Department of Neurology, Hotel-Dieu de France Hospital, Beirut 16-6830, Lebanon; 3 Mellen Center for Multiple Sclerosis, Neurological Institute, Cleveland Clinic Foundation, Cleveland, OH 44195, USA

**Keywords:** multiple sclerosis, disease modifying therapies, safety, long-term outcomes

## Abstract

In the past decade, the therapeutic arsenal for multiple sclerosis has expanded greatly. Newer more potent disease modifying therapies (DMTs) with varying mechanisms of actions are increasingly used early in the disease course. These newer DMTs include oral therapies (teriflunomide, dimethyl fumarate, fingolimod, siponimod, ozanimod, and cladribine) and infusion therapies (natalizumab, alemtuzumab, and ocrelizumab), and are associated with better control of disease activity and long-term outcomes in patients with MS compared to older injectable therapies (interferon beta and glatiramer acetate). However, they are associated with safety concerns and subsequent monitoring requirements. Adverse events are initially observed in phase 2 and 3 clinical trials, and further long-term data are collected in phase 3 extension studies, case series, and post-marketing reports, which highlight the need to periodically re-evaluate and adjust monitoring strategies to optimize treatment safety in an individualized approach.

## 1. Introduction

Multiple sclerosis (MS) is an immune-mediated, chronic inflammatory demyelinating disease of the central nervous system (CNS) affecting over 2 million people worldwide, and a major cause of physical disability in the young [1]. The primary mechanism of CNS injury is demyelination, which is associated with secondary axonal loss and progressive neurodegeneration from the early stages of the disease. Over the last few years, the expansion of the therapeutic arsenal and optimization of MS diagnostic criteria have led to the early use of new disease modifying therapies (DMTs) with varying mechanisms of actions and potentially higher efficacy on the inflammatory component of the disease [2]. As of today, over a dozen DMTs are approved for the treatment of MS, whereas before 2010, only a narrow range of therapeutic options were available: interferons beta, glatiramer acetate, and mitoxantrone, along with off-label medications such as cyclophosphamide. Older first-line therapies in MS include interferon-ß1a, interferon-ß1b, and glatiramer acetate. Along with the newer DMT teriflunomide, a cell-replication inhibitor, DMTs in this category are often referred to as platform therapies, and are considered to have a lower comparative efficacy on controlling disease activity based on their effect on annualized relapse rate and the formation of new lesions on brain magnetic resonance imaging (MRI) in clinical trials. Higher efficacy medications include dimethyl fumarate, an oral immunomodulating therapy, oral (sphingosine-1-phosphate antagonists: fingolimod, siponimod, and ozanimod) and intravenous (natalizumab) inhibitors of cell trafficking, and oral (cladribine) and intravenous (alemtuzumab, and ocrelizumab) cell-deleting therapies. These DMTs are associated with more prominent benefit on both clinical and radiological activity compared to platform therapies.

There are two therapeutic approaches in the current MS DMT era; the escalation approach and the early use of highly effective DMT approach. The escalation approach involves starting with a potentially lower risk, lower efficacy DMT, and switching to a potentially higher risk, higher efficacy DMT when breakthrough disease is observed. For the past 2 decades, MS treatment was based on the concept of therapy escalation [3]. However, this implies using molecules with a lower potential to control inflammation during the first few years, when the inflammatory process of the disease is at its peak. Moreover, injectable therapies, such as interferons and glatiramer acetate, have not been associated with positive outcomes on long-term disability [4]. Early accumulation of physical disability is mostly driven by incomplete recovery from relapses, whereas neurodegeneration plays a more important role in later phases of the disease. For this reason, the early highly effective therapy approach has been increasingly adopted in specialized MS centers. It involves using a higher-efficacy DMT as first-line treatment, early in the disease course. The long-term benefit of newer higher-efficacy DMTs is yet to be elucidated; however, results from recent studies point towards a potential long-term benefit of newer, more potent DMTs, specifically when used earlier in the disease course. In fact, in a large real-world MS cohort, Brown et al. observed a lower likelihood to transition from relapsing remitting MS (RRMS) to secondary progressive MS (SPMS) in patients treated with any DMT in the first 5 years of the disease compared to those who remained off-treatment, and in those treated initially or switched therapies within the first 5 years of the disease with highly effective DMTs compared to those who were on platform therapies [5]. The escalation approach is still widely more accepted because of rising concerns about safety issues and monitoring requirements with newer DMTs, as well as extensive safety experience with platform therapies. Newer DMTs, such as B-cell therapies, cladribine, and alemtuzumab, have long lasting immunosuppressive and immunomodulatory effects, hence the importance of weighing the benefits against the risks in an individualized approach [6].

Older therapies, such as interferon-ß1(a and b) and glatiramer acetate (GA), have been used in the treatment of MS since 1993 and 1998, respectively, and have well-established long-term safety profiles and monitoring strategies based on numerous clinical observations and long-term extension studies. For instance, safety data on GA from an open-label extension study of over 20 years are available [7]. Interferon-ß1’s most common side effects include benign injection site reactions and post-injection flu-like symptoms. Less commonly, lymphopenia, elevation of liver enzymes, headaches, and worsening spasticity and depression can be observed [8]. Exceptionally, congestive heart failure/cardiomyopathy, psychosis, thrombotic microangiopathy, and monoclonal gammopathy. GA most common side effects include injection site reactions, immediate post-injection reactions, and long-term lipoatrophy [9]. Newer versions of interferon-ß1 and GA include generics and pegylated interferon-ß1a (Plegridy^®^, Biogen Idec, Cambridge, MA, USA). The first generic version of GA was approved by the United States Food and Drug Administration (FDA) in 2015 and one year later by the European Medicines Agency (EMA) after favorable results from the GATE trial (Efficacy and Safety of GTR in Comparison to Copaxone, ClinicalTrials.gov NCT01489254) and its extension study showing comparable efficacy, safety profile, and tolerability between brand and generic GA [10]. However, demonstrating equivalence of biologics and biosimilar products to reference drugs is complex, and we are still in the early stages of development of those drugs [11]. Pegylated interferon-ß1a was approved by the FDA for use in RRMS in 2014 after the results of the ADVANCE trial (Pegylated interferon β-1a for relapsing-remitting multiple sclerosis, ClinicalTrials.gov NCT 00906399), which showed similar efficacy and safety compared to the non-pegylated versions of this drug [12]. Overall, older DMTs and their newer versions are considered safe in light of their long track record and evidence from bio-equivalence trials.

In 2006, natalizumab was approved for use in RRMS, followed by fingolimod in 2010, teriflunomide in 2012, dimethyl fumarate in 2013, alemtuzumab in 2014, daclizumab in 2016 (withdrawn in 2017), ocrelizumab in 2017, cladribine and siponimod in 2018, and ozanimod in 2020. The drugs are approved by both of the FDA and the EMA for use in MS to date. The goal of this paper is to review available data on the safety of newer DMTs based on phase 3 clinical trials, trial data from use in other medical conditions, trial data from medications with similar mechanisms, extension studies, case series, and post-marketing reports. We will also discuss different monitoring strategies and suggest supplementary strategies based on an individualized patient-centered approach.

## 2. Safety of Newer DMTs

### 2.1. Safety of DMTs from RCTs, Extension Studies, and Post-Marketing Observations in MS

Regulatory agencies, such as the FDA and the EMA, hold the responsibility to establish protocols to determine the risk-benefit ratio and safety profile of a drug before approval. For instance, the FDA requires adequate and well-controlled investigations encouraging the pharmaceutical companies to develop at least two pivotal trials to prove efficacy and to evaluate short-term safety before approval for use in the general population [13]. These “adequate and well controlled investigations” refer to randomized controlled trials (RCTs), considered to be the gold standard for approving novel drugs [14]. In fact, studies showed that therapies supported by less stringent evidence tend to have higher rates of safety related issues, including boxed warnings and post-marketing reported adverse events [15]. On the other hand, life-threatening adverse events are rare and sometimes cannot be identified during clinical trials. In fact, some serious adverse events are sometimes observed during the post-marketing phase with larger-scale use of the drug on a wider range of patient profiles that are not necessarily represented in RCTs cohorts. Daclizumab is a recent example, as this DMT was withdrawn from the market in 2017 after post-marketing evidence of severe hepatotoxicity and immune central nervous system reactions [16,17]. Fortunately, during the post-marketing phase, data continues to be gathered in extension studies and observational cohorts to establish long-term safety. Additionally, case series and case reports of rare adverse events are reported intermittently, which also contributes to increasing knowledge on safety and periodically reconsider monitoring strategies. In general, the same adverse events observed in RCTs are also observed in manufacturer-sponsored extension studies. Therefore, it is important to periodically review case reports or case series to update DMT safety profiles and identify sub-groups of patients at higher risk of developing certain adverse events. Table 1 lists adverse events reported in phase 3 clinical trials, extension studies, case series, and case reports for newer DMTs used in MS to date.

### 2.2. Safety Experience from DMT Use in Other Conditions

Another mean to identify potential long-term risks of medications is to extrapolate knowledge on safety profile of a molecule when used in a different disease. For example, the hematological experience of cladribine in non-Hodgkin lymphoma and hairy cell leukemia, though used at lower doses than in MS (0.1 mg/kg/day continuous infusion for 7 days) [155] but in a more immunocompromised population, raises a concern of bone marrow suppression with grade 3 and 4 neutropenia, lymphopenia, and thrombocytopenia with development of opportunistic infections like *Pneumocystis carinii* pneumonia and Aspergillosis-related cavitary pneumonia, Candida-associated gastritis, listeriosis, and herpes zoster infections. Other nonhematological adverse events were noted such as alopecia, mucositis, hepatic, and renal toxicities. Decreased left ventricular ejection fraction was also reported [156]. Cases of progressive multifocal leukoencephalopathy (PML) have been reported with cladribine when used for systemic mastocytosis and non-Hodgkins lymphoma [157,158].

Another example would be alemtuzumab, approved in 2001 by the FDA for treatment of B-cell chronic lymphocytic leukemia (B-CLL) [159]. In 2014, the FDA also approved Alemtuzumab for use in MS. Off-label use is also frequent in solid organ transplant especially kidney transplantation [160], although convincing evidence suggest higher rates of virus-associated neoplasms, such as Hodgkin lymphomas, EBV-associated large B-cell lymphoma, Kaposi sarcoma, Human papilloma virus-related cancers, and hepatic carcinoma, in transplant patients who received alemtuzumab in the induction regimen, compared to those who did not [161]. Other complications included CMV reactivation in the first 6 weeks of treatment, pulmonary aspergillosis, HSV/HHV-6 infection, and reactivation of pulmonary tuberculosis [162,163,164]. There was also a report of Guillain–Barré syndrome in a patient on alemtuzumab for T-cell-prolymphocytic leukemia [165,166]. Given the high risk profile of alemtuzumab in terms of safety, the EMA recommended restricting the use of alemtuzumab to patients with highly active RRMS who have failed at least two DMTs [167].

After its approval for MS in 2004, natalizumab was withdrawn from the market after the emergence of reports of PML, conditioning its remarketing in 2006 to a close monitoring of the risks of PML using the Tysabri Outreach Unified Commitment to Health (TOUCH) program developed by the FDA and Biogen-Idec. Natalizumab was also used as the first non-anti-Tumor Necrosis Factor alpha therapy in Crohn’s disease in 2008 [168]. A systematic review published in 2018 showed comparable safety profiles with clinical trials done in MS except new cases of basal cell carcinoma and B-cell lymphoma [122]. Primary central nervous system lymphoma was also reported when used in Crohn’s disease, as well as cases of extrapulmonary sarcoidosis and PML [169,170,171].

### 2.3. Safety Experience from Molecules with Similar Mechanisms of Action

Experience with medications with a similar mechanism of action or a pro-drug also allows anticipating the safety profile of a new molecule. For instance, long-term experience with leflunomide, a prodrug of teriflunomide used in rheumatoid arthritis since 1998, is reassuring except some concerns on dermatological complications (reversible alopecia areata, hidradenitis suppurativa, accelerated nodulosis, and toxic epidermal necrolysis) [172,173,174,175]; a case of colitis with enterocutaneous fistula in a severely immunocompromised patient with concomitant renal transplant, hepatitis E infection in endemic regions, and lymphadenitis and pulmonary infection by *Mycobacterium Paraffinicum* in a patient cotreated with methotrexate [176,177,178].

The chimeric monoclonal antibody rituximab, which has a similar mechanism of action as ocrelizumab, has been used for a wide range of autoimmune diseases for the past couple of decades. It is FDA-approved for use in non-Hodgkin’s lymphoma, chronic lymphocytic leukemia, rheumatoid arthritis, granulomatosis with polyangiitis, microscopic polyangiitis, and pemphigus vulgaris, and has been used off-label in a number of neurological disorders, such as autoimmune encephalitis, myasthenia gravis, neuromyelitis optica spectrum disorders, and more recently, myelin oligodendrocyte glycoprotein (MOG)-related disorders, and other rheumatological and hematological disorders, such as psoriatic arthritis, ankylosing spondylitis, autoimmune hemolytic anemia, autoimmune thrombocytopenia, glomerulonephritis, cryoglobulinemic vasculitis, Sjögren’s syndrome, dermatomyositis, systemic lupus erythematosus, mantle zone lymphoma, and Waldenstrom’s macroglobulinemia. While ocrelizumab was approved for relapsing remitting and primary progressive MS, rituximab has been widely used for the treatment of MS for many years in some countries, particularly Sweden. Rituximab past experience in other auto-immune diseases indicates an increased risk of mild to moderate bacterial infections, except in systemic lupus erythematosus, where many opportunistic infections were noted, such as *Salmonella typhimurium* gastroenteritis, meningococcal meningitis, Listeria meningitis, oesophageal candidiasis, herpes zoster infection and reactivation, endocarditis, septicemia, cholangitis, and *Pneumocystis jirovecii* pneumonia as well as non-infectious complications such as mild to severe lymphopenia, posterior reversible encephalopathy syndrome, unstable angina, and hypogammaglobulinemia [179,180,181,182,183,184,185,186,187]. In lymphoid malignancies, whether used with or without other immunosuppressive medications, important adverse events related to rituximab were tumor lysis syndrome (acute kidney injury, hyperkalemia, hypocalcemia, hyperuricemia, or hyperphosphatemia); and severe mucocutaneous reactions (paraneoplastic pemphigus, Stevens Johnson syndrome, and toxic epidermal necrolysis) [188,189]. Another adverse event worth mentioning is PML. In 2006, many cases of PML were reported in patients treated with rituximab for non-Hodgkin lymphoma. From 1998 to 2017, 734 cases of PML were noted during the post-marketing observation period in patients treated for malignancies such as non-Hodgkin lymphoma and chronic lymphocytic leukemia, and auto-immune diseases like rheumatoid arthritis, granulomatosis with polyangiitis and microscopic polyangiitis. As of October 2019, over 130,000 MS patients have been treated with ocrelizumab. Seven “carry-over” cases of PML were recently reported [141] in patients previously treated with natalizumab or fingolimod, and only one case appears to be attributable to ocrelizumab. There are no reports of PML in other neurological condition such as chronic inflammatory polyradiculoneuropathy, myasthenia gravis, and neuromyelitis optica [190,191,192,193,194,195]. A possible explanation is that cancer patients seem to be more immunocompromised as compared to MS patients due to concomitant use of multiple immunosuppressants, longer exposure with more intensive regimen, and higher frequency of serious adverse events such as leucopenia, a known risk factor for developing PML.

## 3. Monitoring of Adverse Events

A pre-treatment workup should be undertaken before initiating a DMT to ensure patient safety and minimize the risk of serious adverse events through appropriate patient selection. Monitoring adverse events while on therapy is also a major part of DMT management. Recommendations for pre-and post-treatment work-up are typically included in prescribing information sheets provided by the FDA, EMA, and pharmaceutical companies. Table 2 highlights a summary of well-established screening and monitoring recommendations. However, as discussed above, additional safety concerns arise over time, and there is a need to periodically re-consider screening and monitoring strategies based on new evidence. We will discuss examples of safety issues consistently observed with some DMTs that are currently not included in monitoring recommendations. These examples highlight the importance of individualizing screening and monitoring based on current knowledge. An exhaustive list of rare or exceptional adverse events is shown in Table 1.

### 3.1. Cancer Risk Evaluation and Monitoring

Cases of de novo neoplasia have been reported with most DMT in pivotal clinical trials or later on in extension studies or real-world clinical observations (Table 1). In 2011, the EMA refused the marketing authorization of cladribine in relapsing-remitting MS. The potential increased cancer risk was one of the main reasons [213]. However, recent evidence suggests that there is no increased risk in cladribine-treated patients versus placebo based on long term data in pivotal trials [214].

Breast cancer: Rare cases of breast carcinoma have been reported in both ozanimod and ocrelizumab-treated patients in RCTs. There is currently no post-marketing data on ozanimod since it was only approved in 2020. In ocrelizumab-treated patients, malignancies, mainly breast cancer, were found to occur more frequently in pivotal trials. Six out of 781 women treated with ocrelizumab developed breast compared to none in the 668 women treated with interferonß-1b or placebo [215]. The female population risk of breast cancer is estimated at 12% over the course of a lifetime patient is considered at high risk in the presence of one or more of well-established risk factors: family history of breast cancer, personal history of mantle irradiation, personal history of atypical hyperplasia or locular carcinoma in situ, early menarche (<12), late menopause (>55), nulliparity or age at first childbirth >30, hormone replacement therapy with combination estrogen and progesterone, high alcohol intake, obesity and presence of dense breast tissue on mammography [191]. The American College of Radiology, the Society of Breast Imaging, the American Society of Breast Surgeons and the National Comprehensive Cancer Network all agree that women with an average risk of breast cancer should begin yearly screening mammograms at age 40, whereas women deemed to be at higher risk should consider screening with yearly breast MRI starting at age 25 and yearly mammography (3D modality preferred) starting at age 30 [216]. Some MS experts recommend choosing another DMT for women with high risk of breast cancer for whom ocrelizumab is suggested, whereas others initiate ocrelizumab while recommending stringent adherence to screening strategies while on therapy. The VERISMO post-marketing safety study, among other extension studies and observational reports, aims at determining the incidence of malignancies in MS patients treated with ocrelizumab and will help to better evaluate the risk in this population [217].

Melanoma: Melanoma has been reported in MS patients treated with fingolimod [53], and less frequently in patients treated with natalizumab, alemtuzumab and ocrelizumab. Known risk factors for melanoma include a family history of melanoma, the presence of dysplastic nevus, and multiple nevi (≥100). Annual clinical visual skin examination is recommended in high-risk patients and those on high-risk therapies to detect melanoma [218]. The occurrence of aggressive skin cancers, such as melanoma, raises the question of dermatological monitoring. Many experts recommend baseline dermatological examination and self-monitoring thereafter. Others also recommend yearly formal dermatological evaluation while on therapy. In some centers, both pre-and post-treatment skin examinations are not routinely recommended.

### 3.2. HPV-Related Cervical Dysplasia and Cancer

In August 2018, the U.S. Preventive Services Task Force published its final recommendation on cervical cancer screening in average-risk women, which is in line with current cervical cancer screening guidelines from the American College of Obstetricians and Gynecologists, the American Cancer Society, and the American Society for Clinical Pathology; no screening is recommended in women younger than 21 years or older than 65 years with adequate prior screening or in women who have had hysterectomy with removal of the cervix and no prior history of a high-grade cervical precancerous lesion or cervical cancer. As for women aged 21 until 29 years, a cervical cytology is sufficient every 3 years and for those aged 30 to 65 years, either cervical cytology every 3 years, high-risk HPV (hrHPV) testing every 5 years, or both every 5 years are appropriate options [219]. However, clear recommendations on cervical cancer screening among non-HIV immunosuppressed women, (i.e., on DMTs), are still lacking. Annual HPV screening is only recommended in alemtuzumab-treated MS patients, a similar approach to recommended screening in HIV patients. HPV-related benign lesions and cancers are increasingly reported in fingolimod-treated patients, suggesting an increased incidence of skin and mucosal warts with fingolimod [54,55], the majority being HPV-related cervical dysplasia in female patients and one unique case of HPV-related papillary squamous cell carcinoma of the tonsil in male patient [56]. Screening for HPV and cervical cancer is not typically performed as part as routine testing for patient on fingolimod. These recent observations underline the potential need to vaccinate patients for HPV before initiating fingolimod [220]. Recommendations for vaccination in other diseases causing a chronic state of immunosuppression, such as HIV, align with general population guidelines (vaccination between ages 9–26 or before sexual exposure). Since HPV vaccines do not cover all virus subtypes, and patient who are diagnosed with MS are often older than recommended ages of HPV vaccination, screening for HPV-related disease remains the main preventive measure [220]. It is still not clear if conducting yearly Pap smear screening would be useful or cost-effective in female patients on fingolimod or alemtuzumab, but clinician awareness around the possibility of HPV-related cancers is important [220].

### 3.3. Infection Risk Evaluation and Monitoring

Benign infections, especially upper and lower respiratory and urinary tracts infections, have been reported with all DMTs. VZV reactivation risk has been identified in patients with MS on fingolimod and other S1P inhibitors, cladribine, alemtuzumab, natalizumab, and ocrelizumab; however, it is recommended to screen for VZV immunity and vaccinate seronegative patients before initiating fingolimod and alemtuzumab only. Age appropriate vaccination should be undertaken in all patients. Other infections have emerged in the post-marketing period as concerning adverse effects with some DMTs and increasing interest has emerged on the evaluation of infection risk factors with newer DMTs to adapt treatment choice and monitoring accordingly. Some examples are discussed below.

Listeriosis: There have been emerging cases of alemtuzumab-associated listeriosis, which can be life-threatening [95]. At present, the only recommended preventive measures to avoid Listeria infections in MS patients on alemtuzumab consist of avoiding or adequately heating foods that are potential sources of *Listeria monocytogenes* while on therapy. Some experts suggest antibiotic prophylaxis for selected patients. However, currently, there is not enough evidence to back up this recommendation [96].

*Cryptococcal meningitis:* Eight cases of cryptococcal meningitis have been documented in the post-marketing surveillance period of fingolimod in patients with MS [57,58,221,222]. Therefore, CSF analysis with yeast culture and *cryptococcal* antigen detection should be undertaken in fingolimod-treated patients with atypical leptomeningeal enhancement in the appropriate clinical setting [58].

IgG level monitoring and infectious risk: Recent data suggests a relationship between infections and B-cell depleting therapies induced-hypogammaglobulinemia in MS patients [223]. Severe hypogammaglobulinemia is associated with a higher likelihood of severe infections [195,224]. The main predisposing risk factors to the development of hypogammaglobulinemia are low immunoglobulins level at baseline, longer duration of therapy, prior immunosuppression, concomitant use of purine analogues or other immunosuppressive medications, chronic lung or heart disease, and older age [225]. It is unclear if patients who develop early or late hypogammaglobulinemia have an underlying baseline immune dysfunction or if rituximab use causes an acquired secondary immunodeficiency in some individuals [226]. It is also unclear when patients actually develop hypogammaglobulinemia, since depletion of plasmocytes (non-CD20 B cells) theoretically occurs after many years of continuous treatment. This observation is potentially generalizable to MS patients treated with ocrelizumab. A recent study in an ocrelizumab-treated MS cohort showed an association between decreased levels of serum IgM and IgG and an increased risk of infection, specifically in patients with longer disease duration who have been potentially treated with other DMTs in the past and who are older [223]. Moreover, in MS and other neurological, rheumatological, and hematological autoimmune disorders, routine assessment of baseline and on-treatment immunoglobulin level is not recommended. In a large cohort of patients with variable diseases treated with rituximab, around 85% did not have immunoglobulin levels checked before being started on treatment [195]. Detection of hypogammaglobulinemia while on treatment helps identify a subgroup of patients who require close surveillance, consideration of prophylactic antibiotics and/or Ig replacement therapy. This data highlights the potential advantage of monitoring total and specific Ig levels before and during treatment with anti-CD20 drugs to prevent hypogammaglobulinemia–related complications. Some experts suggest checking baseline immunoglobulin levels with B-cell depleting therapies and rechecking them if the patient develops an infection. However, are no clear recommendations to date and most experts proceed on a case-by-case basis.

### 3.4. Monitoring of Treatment Effect and Redosing Adaptation with Cell-Depleting Agents

Cell-depleting therapies are known to have prolonged effects on the immune system. The identification of biomarkers to help evaluate treatment effects over time could enhance monitoring recommendations and optimize treatment dosing schedule. Ideally, using the minimal effective dosing regimen could minimize the risk of treatment-related adverse events without compromising efficacy.

B cell monitoring: CD-20 depleting therapies should be administered before symptoms flare, or theoretically before B cell repopulation. B cell repopulation is closely associated to a recrudescence of disease activity in MS, and other autoimmune diseases [227,228]. Several studies in patients with rheumatoid arthritis have demonstrated that B cell repopulation preceded a rheumatoid arthritis clinical relapse by 4 months. Hence, monitoring of CD19 or CD20+ B lymphocytes counts could serve as a biomarker of treatment efficacy and help predict relapses and optimize infusion schedule [229,230]. In most patients, B-cell repopulation starts 6 months after treatment. Better control of disease activity in patients with rheumatoid arthritis has also been associated to persistent CD27+ memory B cell depletion [231,232,233,234]. The optimal dosing frequency of anti-CD20 therapy is still unclear. Biomarkers such as returning memory CD27+ B cells, or CD19/CD20+ B cells could permit the tailoring of rituximab/ocrelizumab retreatment in an individualized approach, given the variable inter-individual range of B-cell recovery time [235].

T-cell monitoring: During alemtuzumab treatment, absolute lymphocyte counts (ALC) decreases rapidly after the first infusion. B cells return to pre-treatment levels within 3 to 8 months, CD8^+^T within 30 months, whereas CD4^+^T cell can require 2 to 3 years to normalize [236,237,238,239,240]. This lymphocytes reconstitution process is the primary mechanism of treatment effect but is also associated with long-term safety [241]. The optimal dosing frequency of ALC, T cells and/or T cell subtypes with cell-depleting therapies and how to approach severe irreversible lymphopenia is yet to be determined. For patients treated with cladribine, current monitoring strategies recommend ALC dosing before treatment initiation, at month 3, and at month 7 after each course. Anti-herpes prophylaxis with acyclovir is recommended in patients with ALC less than 200 cells per microliter at any time, and close clinical monitoring for infections if ALC is below 500. For alemtuzumab, herpes prophylaxis with acyclovir is recommended for 2 years or until the CD4^+^T cell count is at least 200 cells per microliter. An ongoing multicenter explorative phase IV study [242,243], aims to provide deeper insights into the immunological changes in alemtuzumab-MS treated patients and eventually identify biomarkers for treatment efficacy and safety. 

## 4. Conclusions

In the current MS DMT era, choosing the most appropriate therapy for each patient remains a debatable science. Evidence to support the use of one DMT over another or biomarkers to predict accurately which patient will benefit from the use of a specific medication are lacking. Cohorts represented in RCTs are not always representative of the general population, monitoring while on study is typically performed following a strict process which does not mimic the real world, and head-to-head trials comparing newer DMTs have not been conducted. For these reasons, specialists cannot rely on RCTs to decide which DMT has the most favorable safety and efficacy for each patient. This is reflected in clinical practice guidelines when clear recommendations cannot be formulated if data-driven evidence is insufficient. It is also worth mentioning that clinical trials tend to overlook comorbidities including vascular diseases, mental health problems (up to 60% in a survey of 3884 patients with MS), and other factors (tobacco use, vitamin D deficiencies) which can influence treatment adherence, treatment effects, and safety [244]. In clinical practice, physicians tend to adopt the presumed safer escalation approach, although the early use of high efficacy DMTs is increasing, especially in specialized MS centers. As discussed previously, the escalation approach carries the risk of sub-optimal control of neuroinflammation and subsequent irreversible long-term neurodegeneration [245]. On the other hand, the early use of high efficacy DMTs might offer better long-term outcomes. Decision-making is also influenced by specialists’ individual experiences and patient preference. Recent practice guidelines from the American Academy of Neurology (AAN) and the European Committee of Treatment and Research in Multiple Sclerosis (ECTRIMS)/European Academy of Neurology (EAN) guidelines both recommend DMT initiation at the time of diagnosis in patients with clinically isolated syndrome (patients with their first demyelinating event), encourage early DMT initiation in patients with active RRMS, and support an individualized approach for initial DMT choice by taking into consideration patient characteristics, disease activity, safety, and accessibility to the DMT [246]. The ECTRIMS/EAN guidelines make no recommendation for a specific DMT as first-line treatment for patients with highly active MS, whereas the AAN guidelines recommend using alemtuzumab, fingolimod, or natalizumab [246]. In both guidelines, there is no clear recommendation on specific monitoring strategies, although both panels advise clinical and radiological monitoring, which should be adapted to the DMT and disease severity. Incorporating other types of studies, such as real-world evidence from observational studies and pragmatic trials, into guideline recommendations will be increasingly necessary in the future to address the uncertainty in treatment decision-making [246]. Two pragmatic trials aiming at evaluating the different treatment approaches, “Determining the Effectiveness of early Intensive Versus Escalation Approaches for RRMS” (DELIVER-MS, NCT03535298) and “Traditional Versus Early Aggressive Therapy for Multiple Sclerosis Trial” (TREAT-MS, NCT03500328) are ongoing. The former will evaluate outcomes in treatment-naïve patients started either on moderate-efficacy DMTs versus high-efficacy DMTs, and the latter in patients switching to DMT in the same efficacy category versus a higher-efficacy DMT [247]. Future therapeutic algorithms that will take into account results from these trials, future observational data, and hopefully biomarkers to identify high-risk patients will lead to a personalized treatment approach to maximize efficacy while minimizing risks.

Most of the monitoring strategies are based on phase 3 clinical trials and their extension studies, the longest for the newer DMTs being over 9 years (for teriflunomide [22]). Post-marketing studies suggest a wider and more serious range of adverse events that needs to be taken into consideration when planning future monitoring strategies to prevent unnecessary harm to patients (Table 3). Informing patients about these potential adverse events is essential, as well as establishing a clear monitoring strategy adapted to patient preferences and capabilities. Specialized centers with a multidisciplinary team and comprehensive patient information and education about the disease will help patients and caregivers in optimizing adherence and minimizing adverse events [248].

## Figures and Tables

**Table 1:** Multiple sclerosis disease modifying therapies: adverse events from clinical trials, extension studies, and clinical observations.

DMT, Dosage	Mechanism of Action	Adverse Effects in Multiple Sclerosis Clinical Trials	Adverse Effects in Extension Studies and Case Series/Reports
Oral therapies			
Teriflunomide (AUBAGIO^®^, Sanofi Genzyme, Massachusetts, United States) 7 or 14 mg PO daily	Dihydro-oratate dehydrogenase inhibitor: inhibits of proliferation of auto-reactive T and B cells [18]	TEMSO and TOWER trials: gastrointestinal symptoms, hair thinning, skin rashe, weight loss, headaches, increased blood pressure, infections, increased liver enzymes, pancytopenia, peripheral neuropathies [19,20,21]	TEMSO extension study: results consistent with the core trial, no new AE after 9 years of follow-up [22] TOPIC extension study: no new safety issues [23] Toxic epidermal necrolysis [24]
Dimethyl fumarate (TECFIDERA^®^, Biogen Idec, Massachusetts, United States) 240 mg PO twice daily	- Activates the transcription factor nuclear factor E2-related factor 2 (Nrf2) inducing the expression of endogenous antioxidative factors in vitro - Inhibits the transcription factor nuclear factor κB [25]	DEFINE and CONFIRM trials: Flushing, gastrointestinal symptoms, arthralgia, itching, and lymphopenia [26,27,28]	ENDORSE study: no new AEs after 6 years of follow-up [29] RESPOND study: bacterial sepsis, infectious colitis, pneumonia, urinary tract infection, hypercalcemia, hypokalemia, hydronephrosis, nephrolithiasis, and ovarian cyst rupture [30] 8 cases of PML until the end of 2019 (including one case occurring without lymphopenia), hepatotoxicity [31,32,33,34,35,36]
Fingolimod (GILENYA^®^, Novartis, Basel, Switzerland) 0.5 mg PO daily	Functional antagonist of sphingosine-1- phosphate (S1P) S1P1,3–5: retain circulating central memory T cells and naïve T cells in the lymph nodes [37]	FREEDOMS and TRANSFORMS trials: Lymphopenia, increased blood pressure, macular edema, increased liver enzymes, transient bradycardia and atrioventricular block after the first dose, appendicitis, upper and lower respiratory infections, urinary tract infections, influenza infections, headache, herpesvirus infections, rare cases of fatal herpes simplex encephalitis and disseminated varicella zoster infection, basal-cell carcinoma, malignant melanoma [38,39] FREEDOMS II: results similar to previous trial [40] PARADIGMS (pediatric trial): convulsions, agranulocytosis, arthralgia, autoimmune uveitis, macular edema, bladder spasm, dyspepsia, dysuria, increased liver enzymes, gastrointestinal necrosis (intussusception or necrotic bowel), head injury, humerus fracture, hypersensitivity vasculitis, migraine, migraine without aura, muscular weakness, rectal tenesmus, second degree atrioventricular block, small-intestinal obstruction, leukopenia, appendicitis, cellulitis, gastrointestinal infection, oral abscess, viral infection (pharyngitis) [41]	TRANSFORMS extension study over 4.5 years: similar results to previous trials [42] LONGTERMS: no new or unexpected safety concerns over 14 years of follow-up [43] FREEDOMS extension trial: no new AE over 4 years of follow-up [44] ACROSS study: 10 years follow-up showed no new AE [45] MS NEXT study: no new AE [46] Cryptococcal infections, cryptococcal meningoencephalitis, cryptosporidiosis, visceral leishmaniasis, progressive multifocal encephalopathy, immune reconstitution inflammatory syndrome, human papillomavirus virus-related warts, primary cutaneous Epstein-Barr virus-positive diffuse large B-cell lymphoma, melanoma, flare of hepatitis B virus, varicella-zoster virus vasculopathy, T-cell cutaneous and peripheral T-cell lymphomas, cutaneous anaplastic large cell lymphoma, glioblastoma, Kaposi sarcoma, tumefactive demyelination, necrotizing fungal osteomyelitis [47,48,49,50,51,52,53,54,55,56,57,58,59,60,61,62,63,64,65,66,67,68,69,70,71,72,73,74,75,76,77]
Siponimod (MAYZENT^®^, Novartis, Basel, Switzerland) 2 mg PO daily	Functional antagonist of receptors S1P1 and S1P5 [78]	EXPAND: liver enzyme elevation, bradycardia at treatment initiation, macular edema, nasopharyngitis, hypertension, varicella zoster virus reactivation, and convulsions [79]	No post-marketing data
Ozanimod (ZEPOSIA^®^, Celgene Corporation, New Jersey, United States) 0.5 to 1 mg PO daily	Selective modulator of S1P receptors with improved selectivity for S1P1R and S1P5R [80]	RADIANCE and SUNBEAM trials: nasopharyngitis, headache, hypertension, upper respiratory tract infection, influenza-like illness, back pain, headache, and alanine aminotransferase, liver enzymes elevation, bradycardia at treatment initiation, herpes infections, lymphopenia, macular edema, appendicitis, urticaria, Guillain-Barré syndrome, invasive breast carcinoma, keratoacanthoma, basal cell carcinoma, suicidal ideation [81,82,83]	No post-marketing data
Cladribine (MAVENCLAD^®^, Merck group, Darmstadt, Germany) 3.5 mg/kg over 2 years PO	Synthetic analogue of deoxyadenosine, causes prolonged depletion of circulating T and B lymphocytes [84]	CLARITY and ORACLE-MS studies: Lymphopenia grade 3 and 4, and decreased lymphocyte count, neutropenia, thrombocytopenia, increased blood creatine phosphokinase, herpes zoster infection and reactivation, malignancies (pancreatic carcinoma, ovarian carcinoma, choriocarcinoma reported) Dose related (5.25 mg/kg): lymphopenia, leukopenia, lymphocyte count decreased, vertigo, tinnitus and hypesthesia [85,86]	CLARITY extension study: similar results to previous trials, follow-up up to 4 years [87] Lichenoid rash [88]
Infusion therapies			
Alemtuzumab (LEMTRADA^®^, Sanofi Genzyme, Massachusetts, United States) 12 mg IV/day for 5 consecutive days then 12 mg IV/day for 3 days one year later	Humanized monoclonal antibody selectively directed against the CD52 antigen on T- and B-lymphocytes, causes prolonged depletion of circulating T- and B-lymphocytes [89]	CARE-MS I and II: - Infusion-associated reactions (headache, rash, and pyrexia, non-anaphylactoid hypotension with the first infusion) - Infections: upper respiratory, urinary tract, and herpetic infections, mucocutaneous fungal infections, listeriosis, viral meningitis, tuberculosis - Secondary autoimmune disorders: thyroid disease (hypothyroidism, hyperthyroidism, Graves’ disease, thyrotoxicosis, thyroiditis), renal disease (Goodpasture syndrome, membranous nephropathy), immune thrombocytopenic purpura (ITP, with one case of intracranial bleeding), autoimmune hemolytic anemia - Malignancies (thyroid papillary carcinoma, Burkitt lymphoma) [90,91,92]	CARE-MS I and II extension studies, and TOPAZ studies: Similar AEs over 5 years of follow-up, decreased incidence of infections and thyroid disorders (after the third year) [93,94] Organizing pneumonia, alveolar hemorrhage, myocardial infarction, malignancies (basal-cell carcinoma, malignant melanoma, squamous cell carcinoma, thyroid papillary carcinoma), AV block, ischemic colitis, legionella pneumonia sepsis, opportunistic infections (nocardia, listeria infections, invasive pulmonary aspergillosis, primary Toxoplasma gondii infection, Herpes simplex reactivation, VZV reactivation, and CMV reactivation, mucocutaneous candidiasis), acute acalculous cholecystitis, intracerebral hemorrhage during alemtuzumab administration, cervical artery dissection, hemophagocytic lymphohistocytosis, autoimmune hemolytic anemia, agranulocytosis, autoimmune hepatitis, tumefactive demyelination, acquired hemophilia [95,96,97,98,99,100,101,102,103,104,105,106,107,108,109,110,111,112,113,114,115,116]
Natalizumab (TYZABRI^®^, Biogen Idec, Massachusetts, United States) 300 mg IV monthly	Humanized monoclonal antibody against α4-integrin, inhibits the migration of lymphocytes through the blood-brain barrier into the CNS [117]	AFFIRM, GLANCE, ASCEND, and SENTINEL trials: Infusion reactions, fatigue, headache, arthralgia, hypersensitivity reactions, asymptomatic lymphocytosis, increased liver enzymes, flu-like symptoms, upper respiratory infections, urinary tract infections, anti-natalizumab antibodies, PML [118,119,120,121]	Severe hepatotoxicity in one case, melanoma, primary central nervous system lymphoma, T-cell lymphoma, herpes and varicella-zoster encephalitis and meningitis, cryptococcal meningitis, localized sporotrichosis, tumefactive demyelination [57,122,123,124,125,126,127,128,129,130,131,132,133,134]
Ocrelizumab (OCREVUS^®^, Genentech, California, United States) 300 mg IV 2 weeks apart as initial dose then 600 mg IV every 6 months	Recombinant humanized second-generation anti-CD20 monoclonal antibody [135,136]	OPERA I, OPERA II, and ORATORIO trials: infusion-related reaction (pruritus, rash, throat irritation, and flushing, bronchospasm), nasopharyngitis, upper respiratory tract infection, herpes virus-associated infection, urinary tract infection, pneumonia, aspiration pneumonia, headache, antidrug-binding antibodies, invasive ductal breast carcinoma, renal-cell carcinoma, malignant melanoma, basal-cell skin carcinoma, endometrial adenocarcinoma, anaplastic large-cell lymphoma, malignant fibrous histiocytoma, pancreatic carcinoma, pulmonary embolism, pancreatic carcinoma, reactivation of occult hepatitis B virus infection [137,138,139,140]	One case of late-onset neutropenia associated with mucositis and neutropenic fever, hypogammaglobinemia, DRESS, refractory colitis, aseptic meningitis, endocarditis, Parvovirus B19 infection, tumefactive demyelination, HSV-2-encephalitis, psoriasiform dermatitis, reactivation of hepatitis B virus, fulminant hepatitis associated With Echovirus 25, 8 cases of PML (one of them attributable to ocrelizumab) [141,142,143,144,145,146,147,148,149,150,151,152,153,154]

Abbreviations and identifiers: ACROSS (fingolimod, ClinicalTrials.gov NCT00333138); AE, adverse event; AFFIRM (natalizumab, ClinicalTrials.gov NCT00027300); ASCEND (natalizumab, ClinicalTrials.gov, number NCT01416181); AV, atrioventricular; CARE-MS I (alemtuzumab, ClinicalTrials.gov NCT00530348); CARE-MS II (alemtuzumab, ClinicalTrials.gov NCT00548405); CLARITY (cladribine, ClinicalTrials.gov NCT00213135); CMV, cytomegalovirus; CNS, central nervous system; CONFIRM (*dimethyl fumarate*, clinicaltrial.gov NCT00451451); DEFINE (*dimethyl fumarate*, clinicaltrial.gov NCT00420212); DRESS, drug reaction with eosinophilia and systemic symptoms; ENDORSE (*dimethyl fumarate*, ClinicalTrials.gov NCT00835770); EXPAND (siponimod, ClinicalTrials.gov NCT01665144); FREEDOMS I (fingolimod, ClinicalTrials.gov NCT00289978); FREEDOMS II (fingolimod, ClinicalTrials.gov NCT00355134); GLANCE, Glatiramer Acetate and Natalizumab Combination Evaluation; Hypersensitivity reactions, allergic reaction, an anaphylactic or anaphylactoid reaction, urticaria, or hives; LONGTERMS (fingolimod, ClinicalTrials.gov NCT01201356); MS NEXT (fingolimod, ClinicalTrials.gov); OPERA I (ocrelizumab, ClinicalTrials.gov NCT01247324); OPERA II (ocrelizumab, ClinicalTrials.gov NCT01412333); ORACLE-MS (cladribine, ClinicalTrials.gov NCT00725985); ORATORIO (ocrelizumab, ClinicalTrials.gov number, NCT01194570); PARADIGMS (fingolimod, ClinicalTrials.gov NCT01892722); PML, progressive multifocal leukoencephalopathy; RADIANCE (ozanimod, ClinicalTrials.gov NCT02047734); RESPOND (*dimethyl fumarate*, ClinicalTrials.gov identifier: NCT01903291); S1PR, sphingosine 1-phosphate receptor; SENTINEL (natalizumab, ClinicalTrials.gov NCT00030966); SUNBEAM (ozanimod, ClinicalTrials.gov NCT02294058); TEMSO (*teriflunomide*, clinicaltrial.gov NCT00134563); TOPAZ (alemtuzumab, ClinicalTrials.gov NCT02255656); TOPIC (*teriflunomide*, clinicaltrial.gov NCT00622700); TOWER (*teriflunomide*, clinicaltrial.gov NCT00622700); TRANSFORMS (fingolimod, 1ClinicalTrials.gov NCT00340834); VZV, varicella-zoster virus.

**Table 2:** **FDA** Recommended Pre-Treatment Screening and Monitoring during Treatment with Newer Disease Modifying Therapies.

Disease Modifying Therapy	Routine Screening Prior to Initiation	Routine Monitoring while on Treatment
Oral therapies		
Teriflunomide (AUBAGIO^®^, Sanofi Genzyme, Massachusetts, United States) [22,144,196]	Baseline CBC and LFTs (within 6 months before starting therapy) Latent tuberculosis screening with a tuberculin skin test. Baseline blood pressure Pregnancy test, counseling on use of effective contraception in males and females of reproductive potential	CBC if signs/symptoms of hematologic toxicity, LFTs monthly for 6 months, then every 6 months thereafter Periodic blood pressure monitoring Confirm use of effective contraception at each encounter for both male and female patients during therapy
Dimethyl fumarate (TECFIDERA^®^, Biogen Idec, Massachusetts, United States) [144,197,198]	Baseline CBC with absolute lymphocyte count Baseline LFTs and renal function tests Pregnancy test, counseling on use of effective contraception in females of reproductive potential	CBC with lymphocyte count after 6 months, every 6 to 12 months thereafter or as clinically indicated LFTs and renal function tests after 3 and 6 months, every 6 to 12 months thereafter and as clinically indicated Confirm use of effective contraception at each encounter for female patients during therapy
Fingolimod (GILENYA^®^, Novartis, Basel, Switzerland) [37,42,199,200,201,202]	Baseline CBC and LFTs (within 6 months before starting therapy) Baseline ECG, review of medications (concomitant use of medications causing bradycardia or conduction abnormalities) Baseline ophthalmologic examination (or optical coherence tomography) Baseline skin examination (in most centers) Pregnancy test, counseling on use of effective contraception in females of reproductive potential VZV serology or confirmation of prior exposure. VZV vaccine in non-immunized patients prior to treatment start. Baseline clinical evaluation of respiratory function	CBC after 1, 3 and 6 months and periodically thereafter. LFTs after 1 month then every 3 months for the first year, then periodically thereafter First-dose cardiac monitoring Periodic blood pressure monitoring Ophthalmologic examination (or optical coherence tomography) 3–4 months after initiation, and at any time if patient reports visual disturbances. Regular ophthalmologic follow-up in patients with diabetes mellitus or a history of uveitis Self-check or dermatologist examination annually (in many centers) Confirm use of effective contraception at each encounter for female patients during therapy
Siponimod (MAYZENT^®^, Novartis, Basel, Switzerland) [203]	CYP2C9 genetic testing Baseline ECG. Baseline blood pressure Baseline ophthalmologic examination Baseline CBC and LFTs (within 6 months before starting therapy) Pregnancy test, counseling on use of effective contraception in females of reproductive potential VZV serology or confirmation of prior exposure. VZV vaccine in non-immunized patients prior to treatment start Baseline evaluation of respiratory function	First-dose observation in high-risk patients only. Periodic blood pressure monitoring Ophthalmologic examination if clinically indicated CBC and LFT 3–6 months after starting treatment, then every 6–12 months Confirm use of effective contraception at each encounter for female patients during therapy
Ozanimod (ZEPOSIA^®^, Celgene Corporation, New Jersey, United States) [46,204,205]	Obtain CBC and LFTs (within 6 months before starting therapy) Baseline ECG and baseline blood pressure Baseline ophthalmologic examination Pregnancy test, counseling on use of effective contraception in females of reproductive potential VZV serology or confirmation of prior exposure. VZV vaccine in non-immunized patients prior to treatment start. Baseline evaluation of respiratory function	CBC and LFT 3–6 months after starting treatment, then every 6–12 months Periodic blood pressure monitoring Ophthalmologic examination id clinically indicated onfirm use of effective contraception at each encounter for female patients during therapy
Cladribine (MAVENCLAD^®^, Merck group, Darmstadt, Germany) [206]	CBC including absolute lymphocyte count and LFTs before each dose Pregnancy test, counseling on use of effective contraception in females and males of reproductive potential for at least 6 months after each dose Baseline Antibodies to VZV; VZV vaccination in antibody-negative patients 1 month prior to treatment initiation Tuberculosis screening, Hepatitis B and C panels, and HIV serology Standard age-appropriate cancer screening recommendations	CBC at 2 and 6 months after each dose Confirm use of effective contraception at each encounter for female patients during therapy Anti-herpes prophylaxis with acyclovir in patients with ALC less than 200 cells per microliter, and close monitoring for infections if ALC is below 500 cells per microliter
Infusion therapies		
Natalizumab (TYSABRI^®^, Biogen Idec, Massachusetts, United States) [84,126,128,207,208,209,210]	Anti-JCV antibody serostatus and index Baseline CBC and LFTs Obtain a recent brain MRI (usually within 3 months)	Anti-JCV antibody testing every 6 to 12 months in seronegative patients for the first 2 years, and every 3–6 months thereafter CBC and LFT after 1, 3 and 6 months and every 6 months thereafter. Brain MRI yearly in JCV seronegative patients for the first 2 years, and every 3–6 months thereafter depending on JCV serostatus and risk of PML Neutralizing antibodies to natalizumab 6 months after initiation or at any time in patients with hypersensitivity reactions or disease exacerbations while on therapy.
Alemtuzumab (LEMTRADA^®^, Sanofi, Genzyme, Massachusetts, United States) [116,211]	CBC, TSH, LFTs, creatinine, urine analysis with cell counts and urine protein to creatinine ratio Baseline skin (melanoma screening) and gynecologic (HPV/cervical dysplasia screening) exam Pregnancy test, counseling on use of effective contraception in females of reproductive potential for at least 4 months after each cycle Tuberculosis screening, Hepatitis B and C panels, and HIV serology VZV serology or confirmation of prior exposure. VZV vaccine in non-immunized patients prior to treatment start. Counselling on Listeria monocytogenes infection risk 2 weeks prior treatment initiation and while on treatment (avoidance of undercooked deli meat, seafood, or poultry, dairy products made with unpasteurized milk, and soft cheeses)	TSH every 3 months for 48 months following last course (second or subsequent course) CBC, creatinine and urine analysis with cell counts monthly for 48 months following last course (second or subsequent course). LFTs periodically until 48 months after the last dose Anti-viral prophylaxis with acyclovir 200 mg p.o twice daily for 24 months starting day 1 of first cycle, or for continue for a minimum of two months following treatment or until the CD4+ lymphocyte count is at least 200 cells per microliter, whichever occurs later Perform yearly skin and gynecologic exam Confirm use of effective contraception at each encounter for female patients during therapy
Ocrelizumab (OCREVUS^®^, Genentech, California, United States) [212]	Baseline CBC and LFTs Hepatitis B and C and Tuberculosis screening. Pregnancy test, counseling on use of effective contraception in females of reproductive potential for at least 2 months after each cycle Vaccines according to age-appropriate immunization guidelines at least 6 weeks prior to treatment start Age-appropriate breast cancer screening	CBC and LFTs every 6 months Immunoglobulin levels in patients with severe or recurrent infections Confirm use of effective contraception at each encounter for female patients during therapy

Abbreviations: ALT: alanine aminotransferase; AST: aspartate aminotransferase; CBC: complete blood count; ECG: electrocardiogram; HBV: Hepatitis B virus; HCV: Hepatitis C virus; HIV: Human Immunodeficiency Virus; LFT: liver function test; TSH: Thyroid-stimulating hormone. CBC and LFT dosage with oral medications are checked every 3–6 months depending on practice and patient profile.

**Table 3:** Suggested additional monitoring strategies with newer disease modifying therapies.

Disease Modifying Therapy	Suggested Strategies
Fingolimod (GILENYA^®^, Novartis, Basel, Switzerland)	Systematic pre-treatment and annual formal skin examination thereafter in all patients Gynecologic (HPV/cervical dysplasia screening) exam according to guidelines, consider yearly evaluation in high-risk patients, pre-treatment HPV vaccination as appropriate for age Brain MRI with contrast and CSF analysis with cryptococcal testing in the appropriate clinical context
Cladribine (MAVENCLAD^®^, Merck group, Darmstadt, Germany)	Consider periodic lymphocyte phenotyping and close monitoring for infectious risk accordingly
Alemtuzumab (LEMTRADA^®^, Sanofi, Genzyme, Cambridge, MA, USA)	Consider periodic lymphocyte phenotyping and close monitoring for infectious risk accordingly
Ocrelizumab (OCREVUS^®^, Genentech, South San Francisco, CA, USA)	Breast cancer risk stratification and screening/monitoring accordingly. Pre-treatment and periodic immunoglobulin G, M and A levels CD19/CD20+ B cells levels for dosing adaptation
